# Peritoneal and genital coccidioidomycosis in an otherwise healthy Danish female: a case report

**DOI:** 10.1186/s12879-017-2212-4

**Published:** 2017-01-31

**Authors:** Ole Bæk, Karen Astvad, Reza Serizawa, Lawrence J. Wheat, Pia T. Brenøe, Ann-Brit E. Hansen

**Affiliations:** 1 Department of Infectious Diseases, Copenhagen University Hospital, Hvidovre, Denmark; 2 Department of Microbiology and Infection Control, Statens Serum Institut, Unit of Mycology, Copenhagen, Denmark; 3 Department of Pathology, Copenhagen University Hospital, Hvidovre, Denmark; 4 MiraVista Diagnostics, Indianapolis, IN USA; 5 Department of Gynecology, Copenhagen University Hospital, Hvidovre, Denmark

**Keywords:** Case report, Coccidioidomycosis, Disseminated, Fungus, Traveller

## Abstract

**Background:**

Coccidioidomycosis is a fungal infection that usually presents as a primary lung infection. The fungus is endemic to the Southwest United States of America, northern Mexico and parts of Central and South America the infection is rare outside these areas. However, some patients develop disseminated infection that can lie dormant for several years and can present itself in travelers. We report the first case of extra pulmonary Coccidioidomycosis in a non-immunocompromised individual in Denmark.

**Case presentation:**

A 32 year old Danish woman presented at the Emergency department with abdominal pain. Computed tomography scan and ultrasound examination of the pelvis raised suspicion of salpingitis. A laparoscopy exposed a necrotic salpinx and several small white elements that resembled peritoneal carcinomatosis. Histological workup however determined that she suffered from disseminated coccidioidomycosis. The patient had lived 2 years in Las Vegas, in the United States of America, 7 years prior and had no memory of lung infection at the time.

**Conclusions:**

Disseminated coccidioidomycosis is rare in non-immunocompromised individuals. The patient in this case underwent several rounds of in vitro fertilization treatment in the years before admittance. We suspect that the hormonal treatment in combination with low-dose prednisolone may have triggered reemergence of the disease and present literature that support this.

## Background

Coccidioidomycosis (CM) is a fungal infection caused by infection by either *Coccidioides immitis* or *C. posadasii*. The two species are closely related and morphologically identical [1]. The fungi are endemic to the arid desert areas of the southwest United States of America (USA) and northern Mexico, but can also occasionally be found in other desert areas in South America. The fungi are dimorphic, with the mycelial (mold) stage growing in dry soil. During growth the mycelium breaks down into separate arthroconidia, which are very small (5 × 4 um) and easily carried away on dust particles. When inhaled the tiny arthroconidia reach the alveoli where they develop into the endospore (parasitic) stage. This causes a primary lung infection that in most cases is self-limiting. In 5% of patients the primary infection may be followed by the development of pulmonary nodules, cavities, or progressive pulmonary disease. In rare cases patients may develop progressive disseminated disease at the time of initial presentation or years later, presumably as a result of reactivation of latent infection. Disseminated disease is seen in up to 1% of cases and often in immunocompromised individuals and those of African American or Filipino descent. The spread is hematogenous and the most common sites of secondary infection are the meninges, skin, joints and bones. Disseminated disease is associated with a poor prognosis, high mortality and may require lifelong anti-fungal treatment [2, 3].

The diagnosis is based on histopathological demonstration of fungal structures, culture (Biosafety level 3), skin tests, demonstration of humoral antibodies (with commercially available methods of enzyme immune assay (EIA) and immunodiffusion (available for both early IgM and late IgG) and complement fixation (IgG), a quantitative antigen detection EIA and newer molecular methods such as polymerase chain reaction for coccidioidal DNA [2, 3].

Localized extra-pulmonary infection also occurs in immuno-competent individuals. We report an unusual presentation of CM in a young Danish woman diagnosed after being admitted to hospital for acute abdominal pain.

## Case presentation

A 32-year-old Caucasian female presented to the emergency department in 2015 complaining of abdominal pain, with acute onset 8 h prior. The pain was constant, localized in the right lower quadrant and intensity was rated as 8–9 on the visual analogue scale. Routine biochemistry including leukocyte count and C-reactive protein was normal.

A computed tomography (CT) scan of the abdomen revealed fluid in the peritoneum and possible cysts on the right ovary. A gynecological examination was normal, aside from tenderness behind the uterus. A transvaginal ultrasound showed free fluid in the Pouch of Douglass and a 5x4x6 cm large mass between the right ovary and the uterus. At this point the pain had improved considerably on pain medication and the patient was scheduled for an explorative laparoscopy 4 days later.

The laparoscopy found a dilated, necrotic and torqued right salpinx, congealed blood in the Pouch of Douglass and several 1–2 mm, white, round elements on both the salpinx and on all peritoneal surfaces. The right salpinx and several of the peritoneal elements were sent for histological examination alone. Due to the macroscopic appearance, peritoneal carcinomatosis was suspected, although CT scan and laparoscopy had not exposed any underlying malignancies.

**Fig. 1:**
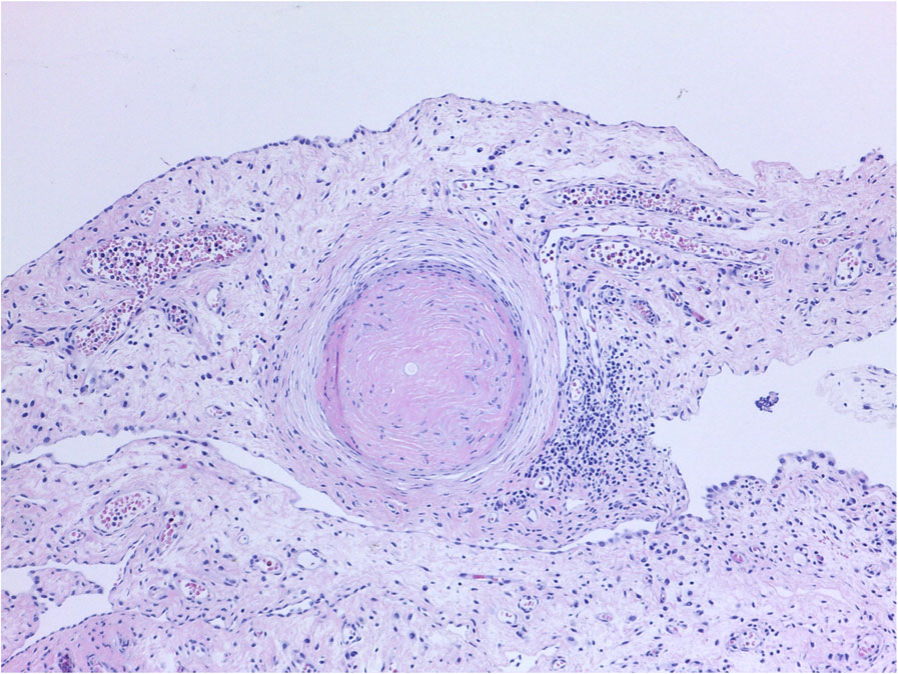
Peritoneal biopsy showing a small occluded vessel with a spherule (original magnification × 100, HE-stain)

**Fig. 2:**
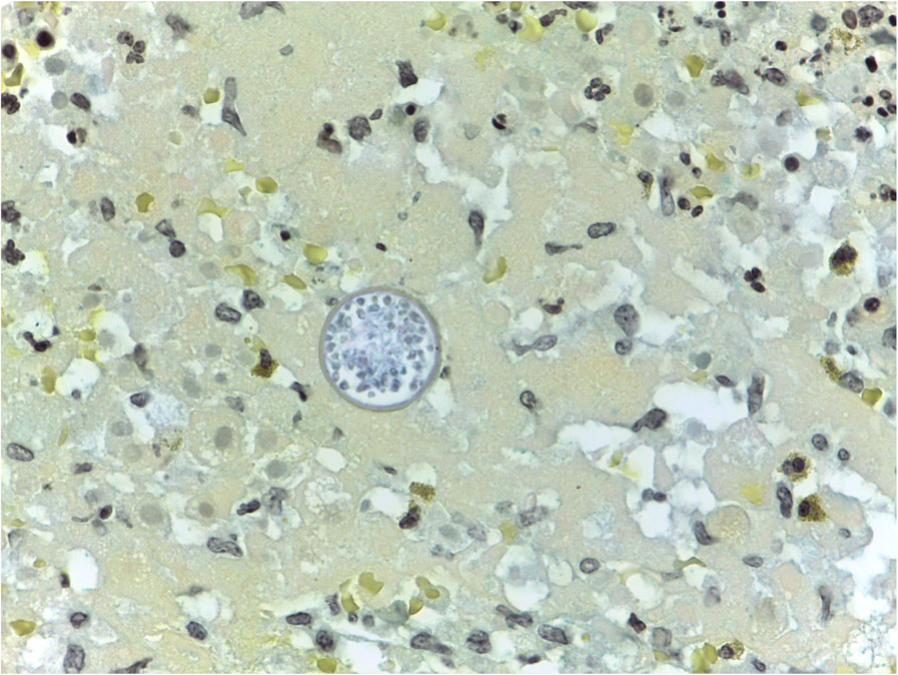
30 μm large endospore-containing spherule, floating in necrotic debris (original magnification × 630, AV-stain)

However, histological examination revealed no signs of malignancy, but a dilated salpinx with fungal structures, widespread necrosis and severe inflammation with numerous eosinophilic granulocytes and vasculitis like changes in the sub serosal vessels. The peritoneal biopsies showed small thrombotic vessels with fungal structures, within the thrombotic material (Fig. 1). The pathologist suspected blastomycosis or another dimorphic fungus. The final histopathological diagnosis of CM was made by identification of the typical large (10–100 μm) endospore-containing spherulae [4] after consulting Danish (Statens Serum Institut) and American microbiologists (Indiana Health Pathology, Indiana University Medical Center, Indianapolis, Indiana, USA) (Fig. 2). No unfixed tissue samples were available for culture or molecular identification methods.

The patient was referred to the department of infectious diseases where further history taking revealed that the patient had lived in Las Vegas, Nevada from 2006 to 2008 and revisited in 2009 and 2011. In the last 5 years she has also visited Argentina, Guatemala, Nigeria, Zambia and South Africa. She could not recall any episodes of illness while traveling. The diagnosis was confirmed by serology [5, 6] (immunodiffusion (Meridian Bioscence, OH, USA)) demonstrating IgG antibodies against *Coccidioides*, with an antibody titer of 1:8. Further workup with a CT-scan of the thorax showed a 16 mm large calcified process in the apex of the right lung. There were no signs of immune deficiency with a negative HIV-test, normal levels of immunoglobulins and a normal CD4 cell count of 1020 cells/μL. Her medical history was inconspicuous; the only prior hospital contact was for fertility treatment that did not result in pregnancy. One to 3 years prior to admittance she underwent four separate in vitro fertilization (IVF) treatments. For the last two rounds of IVF she was also treated with a low dose of prednisolone, 10 mg daily. In 2012, three days after the first insemination, the patient was admitted to the ED for abdominal pain, was observed for 24 h and discharged without further workup.

**Fig. 3:**
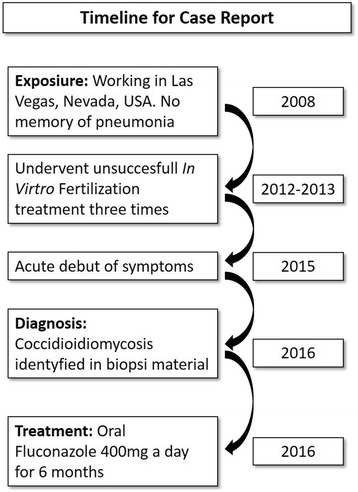
Timeline of case report

The patient was started on treatment with oral fluconazole 400 mg twice daily for 1 week, followed by 400 mg once daily. Treatment was scheduled for at least 6 months and possibly longer guided by serological response. Three months and 6 months after initiation of treatment the patient’s antibody titer had fallen to 1:2 and 1:1 respectively. The patient completed 8 months of fluconazole treatment, with a reduced dose of 200 mg daily for the last month. Two months after completion of therapy the antibody titer was still 1:1. To ensure that the pelvic and peritoneal CM had resolved a laparoscopy was done three and a half month after completion of therapy. The laparoscopy found a dilated, chronic inflamed left salpinx and persistence of the small 1–2 mm, white, round elements on the peritoneal surfaces. The histological examination confirmed granulomatous infection with numerous coccidioidal spherules. The patient was immediately restarted on treatment with oral fluconazole 400 mg twice daily. At the moment, length of treatment has not been decided and other treatment options, such as liposomal amphotericin-B or voriconazole, are being considered. The timeline for the case is summerized in (Fig. 3).

## Discussion

The peritoneum and genital tract are rare sites of extra pulmonary CM. A review from 2015 identified 34 case reports of peritoneal involvement, mostly male (76%) living in endemic areas [7]. As for genital involvement, a review from 1999 found 13 cases of either tubo-ovarian or endometrial disease [8]. The patient in our case showed no signs of systemic disease. Other cases have been reported where patients have localized extra pulmonary CM without systemic disease [9–11]. It is probable that the patient had a primary lung infection while living in the USA that was either asymptomatic or that she could not recall. The process found in the lung could be a remnant of this. The diagnosis of this clinical case was done by histopathological identification of typical spherules, thus we cannot determine whether the infection was caused by Coccidioides immitis or Coccidioides posadasii. The diagnosis was not confirmed by culture or by molecular methods. However, it is recognized that the diagnosis of disseminated coccidioidomycosis can rely on the histopathologic identification of fungal structures in a sample from an extrapulmonary lesion [3], further the diagnosis was corroborated serologically and consistent with the patient’s travel history.

The initial treatment course was for 8 months. But despite the clinical well-being of the patient and a marked serological response, the laparoscopy revealed persistent peritoneal infection and that the coccidioidal infection had spread to the left salpinx. We cannot determine whether the coccidioidal infection had never been cleared or whether the infection had relapsed when fluconazole was discontinued. The Infectious Diseases Socieaty of America (IDSA) guidelines recommend at least 6–12 months of treatment for extrapulmonal soft tissue infection [3] and our case emphasizes the importance of long-term treatment.

In our case, the peritoneal plaques were initially suspected to represent peritoneal carcinomatosis. In two previously published cases, female genital coccidioidomycosis mimicked ovarian cancer due to the presence of an ovarian mass in combination with elevated levels of the tumor-markers CA125 and CA 19–9 [12, 13] and malignant-appearing nodules on all serosal surfaces of the peritoneum [13]. Pregnancy is a known major risk factor for disseminated disease. The reasons are thought to be a combination of the mild immune suppression that occurs during pregnancy, combined with the fact that sex-hormones seems to aid dissemination [14]. Especially high levels of progesterone and 17β-estradiol have been shown to stimulate growth of *C. immitis* in vitro [15].

The patient in our case was not immunocompromised or pregnant. As mentioned, she did however, undergo four separate IVF treatments 1–3 years before admittance. Prior to each treatment the patient was given clomiphene to initiate controlled ovarian stimulation, during which estradiol levels rise. It is possible that the IVF treatments, perhaps in combination with mild immunosuppression caused by the concurrent prednisolone treatment, could have triggered dormant stages of the disease and that dissemination may possibly have happened as far back as 2012. We have not been able to identify other cases in the literature where IVF has been associated with CM. It is also possible that a latent chronic coccidioidal salpingitis could be the primary cause of the patient’s infertility. A long history of infertility has also been reported in other women diagnosed with genital CM [16].

## Conclusion

Imported CM is a rare condition in Denmark, with only one case diagnosed approximately every 10 years [17, 18]. This is the first reported case of extra pulmonary CM, involving the peritoneum and genital tract, in an otherwise healthy individual in Denmark. The case shows that physicians outside endemic areas have to be aware of this disease that can reemerge several years after exposure. The case history also reveals a possible connection with prior IVF treatment, which could have triggered latent chronic infection. This has to our knowledge not been reported anywhere else.

## Abbreviations

CM: Coccidioidomycosis
CT: Computed Tomography
IVF: In vitro fertilization
USA: United States of America
